# Supplementary material for: Vitamin D Modified DSS-Induced Colitis in Mice via STING Signaling Pathway
Source: Biology (Basel). 2025 Jun 18;14(6):715. doi: 10.3390/biology14060715 (PMC12190092; doi:10.3390/biology14060715)
Supplement: Supplementary file 1 [file biology-14-00715-s001.zip › Supplementary Materials S1: 16s-report of gut microbiota/Groups/diff_analysis/TukeyHSD/Tukey_Genus.pdf]

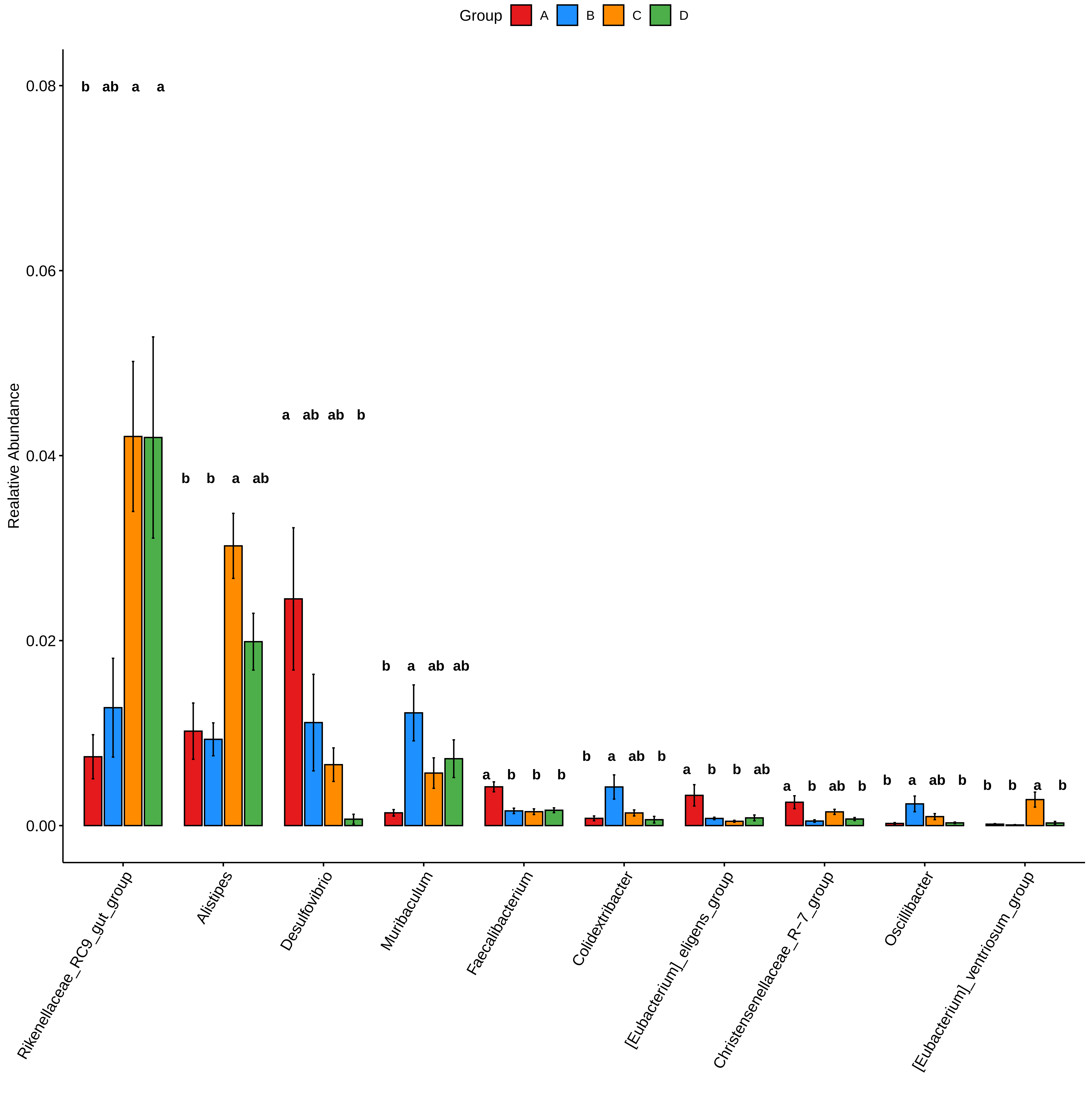

Group
A
B
C
D
Realative Abundance
0.08
0.06
0.04
0.02
0.00
b ab a a
b b a ab
a ab ab b
b a ab ab
a b b b
b a ab b
a b b ab
a b ab b
b a ab b
b b a b
Rikenellaceae_RC9_gut_group
Alistipes
Desulfovibrio
Muribaculum
Faecalibacterium
Colidextribacter
[Eubacterium]_eligens_group
Christensenellaceae_R−7_group
Oscillibacter
[Eubacterium]_ventriosum_group
